# Electrical Brain Responses in Language-Impaired Children Reveal Grammar-Specific Deficits

**DOI:** 10.1371/journal.pone.0001832

**Published:** 2008-03-12

**Authors:** Elisabeth Fonteneau, Heather K. J. van der Lely

**Affiliations:** UCL Centre for Developmental Language Disorders and Cognitive Neuroscience, University College London, London, United Kingdom; Harvard University, United States of America

## Abstract

**Background:**

Scientific and public fascination with human language have included intensive scrutiny of language disorders as a new window onto the biological foundations of language and its evolutionary origins. Specific language impairment (SLI), which affects over 7% of children, is one such disorder. SLI has received robust scientific attention, in part because of its recent linkage to a specific gene and loci on chromosomes and in part because of the prevailing question regarding the scope of its language impairment: Does the disorder impact the general ability to segment and process language or a specific ability to compute grammar? Here we provide novel electrophysiological data showing a domain-specific deficit within the grammar of language that has been hitherto undetectable through behavioural data alone.

**Methods and Findings:**

We presented participants with Grammatical(G)-SLI, age-matched controls, and younger child and adult controls, with questions containing syntactic violations and sentences containing semantic violations. Electrophysiological brain responses revealed a selective impairment to only neural circuitry that is specific to grammatical processing in G-SLI. Furthermore, the participants with G-SLI appeared to be partially compensating for their syntactic deficit by using neural circuitry associated with semantic processing and all non-grammar-specific and low-level auditory neural responses were normal.

**Conclusions:**

The findings indicate that grammatical neural circuitry underlying language is a developmentally unique system in the functional architecture of the brain, and this complex higher cognitive system can be selectively impaired. The findings advance fundamental understanding about how cognitive systems develop and all human language is represented and processed in the brain.

## Introduction

Grammar is an exclusively human and complex ability[1], [2], yet by age 3 years, most children produce grammatically correct sentences. We still have little understanding of the biological and evolutionary changes that enable humans to do this or the changes that prevent them doing this normally as is found in children with SLI, who continue to make grammatical errors, sometimes into adulthood[3].

SLI variably affects the acquisition of subsystems or “components” of language[3]; that is both grammatical components such as syntax (the structural rules combining words into sentences); morphology (the rules combining words or parts of words into new words, e.g., jump+ed); and phonology (the rules for combining sounds into words); word-storage (vocabulary) and other aspects of the conversational (discourse) and social use (pragmatics) of natural language.

The discovery of the subgroup Grammatical(G)-SLI provides rare insight into the neural systems in the human brain and thus its nature and origins are hotly debated. The controversy surrounding G-SLI focuses on whether it results from a domain-general deficit in auditory processing speed or capacity[4], [5], or whether it results from a developmentally specialised grammatical subsystem in the brain that can be selectively impaired[3]. Preliminary evidence from G-SLI reveals familiar clustering of language impairment that is consistent with an autosomal dominant inheritance[6]. However the nature of the language impairment in family members varies, suggesting a more complex inheritance[6]. The G-SLI impairment is life-long, and affects grammatical rules underlying structures in syntax, morphology, and phonology[3]. G-SLI teenagers make errors that normally-developing children rarely make after 5 years of age. For example, they make errors in knowing who *him* or *himself* refers to in the sentence *Mowgli said Baloo was tickling* ***him/himself*** *,* or produce errors when asking questions (*Who* ***__*** *Joe see* ***someone*** *?)* [3]. In contrast, individuals with G-SLI show good understanding of social and world knowledge when they communicate[7], do not show any consistent auditory deficits[8] (see supporting Data S1, Table S1 and Figures S1 and S2) and are of average intelligence[3], [7]. However, behavioural data alone cannot tell us whether the deficit is restricted to only grammar or impacts on more general language–related processing.

Electrophysiological measurements provide direct assessment of brain activity and have the necessary temporal resolution to distinguish between the two hypotheses: generally slow auditory and language mechanisms[5] versus a selective impairment in grammatical mechanisms alongside normal functioning in other language mechanisms[3]. Such residual normality is claimed not to exist[9]. Specifically, electrophysiological, event-related measurements can differentiate neural systems that appear to be automatic, fast, and specific to only grammatical (syntactic) processing (“Early Left-Anterior Negative electrical brain response around 100 ms (ELAN)[10], from systems associated with language processing but which are not grammar-specific, such as an anterior or central positive electrical brain response around 600 ms (P600), often associated with structural syntactic re-analysis of sentences[10], [11] and a posterior negative electrical response around 400 ms (N400) associated with semantic processing[12]. Importantly, whereas the P600 is elicited by a range of different grammatical violations[13] as well as semantic violations[14], the ELAN is only elicited by structural grammatical violations[10]. These differences allow us to make clear predictions for G-SLI. Whereas domain-general hypotheses predict that most if not all ERP language-related components will be affected (e.g., delayed latency), the domain-specific hypothesis predicts that only the grammar-specific component (ELAN), that reflects pure syntactic structure[10], will be atypical or absent.

To investigate these alternative hypotheses we recorded electrophysiological time-locked, event-related brain potentials (ERPs) in 18 participants ages 10 to 21 years, age-matched controls, and younger child and adult controls listening to questions containing a syntactic violation (Experiment 1) (see materials and methods). The particular syntactic violation we were interested in concerns structural “syntactic dependencies” such as those that occur between a question word (*who, what)* and the word, that in declarative sentences follows the verb, but typically is absent in questions (see supporting Methods S1). Such syntactic dependencies make sentences such as “*Who did Joe see someone*?” ungrammatical, but “*Who did Joe see ?”* and *“Joe saw someone”* grammatical. We hypothesised that G-SLI children's syntactic impairment lies in the computational grammatical system underlying such syntactic dependencies[3].

## Results

**Figure 1:**
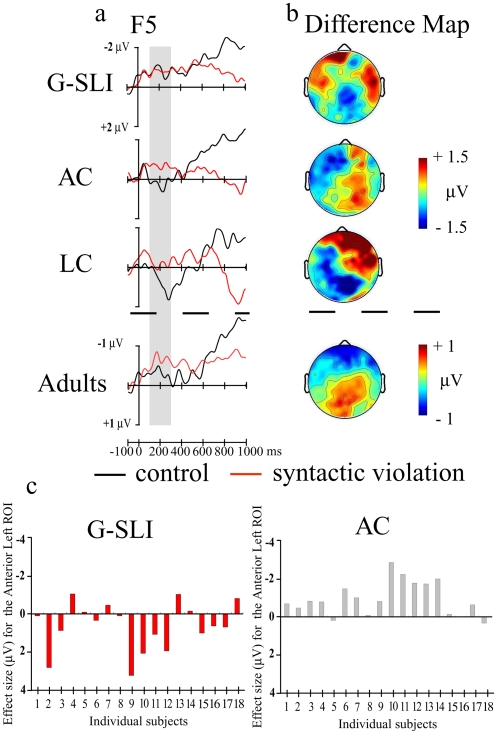
Syntactic dependency component (ELAN, 100–300 ms -grey area) elicited for the syntactic violation. *a.* ERP waveforms for the groups from F5 (left frontal) electrode. *b.* Scalp distribution of differences between the violation minus control sentences for each group. The syntactic violation elicited a negativity distributed on the left hemisphere for the age controls (Condition×Hemisphere: *F*_1,17_ = 10.16, *p*<.005), and the language controls (Condition×Hemisphere: *F*_1,19_ = 11.12, *p*<.003; Condition×Caudality×Hemisphere: *F*_2,38_ = 3.55, *p*<.05), with a maximum of difference on the anterior left sites for both groups (*p*<.006, *p*<.05 respectively). This negativity is equally distributed on anterior sites for the adults (Condition×Caudality: *F*_2,38_ = 10.17, *p*<.001; anterior central *p*<.001). No effect was significant for the G-SLI children (*F*<1). *c.* Effect sizes for individual G-SLI children and their age controls (numbers correspond to matched individuals with increasing numbers corresponding to increasing age). Effect size: mean amplitude difference (violation minus control) in the Anterior Left ROI in the 100–300 ms time window. We plot Negativity upward.

First, we analysed ERPs in the time window 100–300 ms (Figure 1) to assess participants' automatic brain responses to the structural syntactic violations (see methods, and supporting Methods S1 and supporting data in Table S1, and Figure S4). The ERPs for the G-SLI group were compared with those of the age and language controls. The overall ANOVA revealed a group×condition×region of interest (ROI) interaction (*F*_16, 424_ = 1.82, *p*<.027). The syntactic violation elicited an Early Left Anterior Negativity (ELAN) in the age and language controls, which was absent in the G-SLI group (Figure 1a). Individual subject analysis revealed that whereas almost all the age control subjects revealed an ELAN, the G-SLI subjects did not (Figure 1c). A similar brain response, distributed equally on anterior sites was found in our adults (Figure 1b) and, previously, has been elicited in young children, some under 3 years old[15], [16]. The ELAN is considered to be a brain correlate of automatic syntactic structural building and processing[10] and thus, core to the syntactic system[2]. Moreover, the ELAN's sensitivity appears domain-specific to syntactic structure. It is insensitive to task demands or violation frequency that incur other cognitive processes[17], [18]. Thus, our pattern of results is exactly what would be expected if G-SLI children were impaired in a specific mechanism underlying grammar.

**Figure 2:**
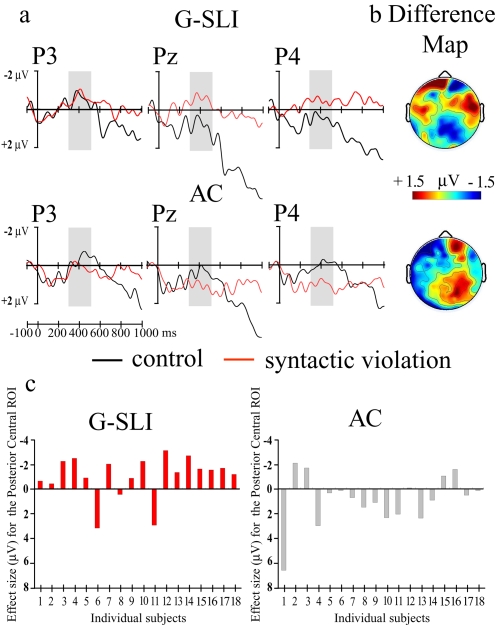
Semantic component (N400, 300–500 ms –grey area) elicited for the syntactic violation for the G-SLI group. *a.* ERPs from three posterior electrodes (P3, left P4 right hemisphere and Pz midline) for the G-SLI and Age Control groups. *b.* Scalp distribution of the differences between the violation minus control sentences. The ERPs from the G-SLI participants elicited a negativity distributed on the posterior area for the syntactic violation (Condition×Caudality: *F*_2,34_ = 3.08, *p*<.05). Note the raw data suggested a lateralisation of the N400 (Condition×Caudality×Laterality *F*_2,34_ = 3.75, *p* = .03) whereas the normalised data indicated a non significant interaction (*F*_2,34_ = 1.93, *p* = .15). No other group showed this result for the 300–500 ms time window. *c.* Effect sizes for individual G-SLI children and their age controls. Effect size: mean amplitude difference (violation minus control) in the Posterior Central ROI within the 300–500 ms temporal window. We plot Negativity upward.

To test the hypothesis that our G-SLI children were “slow processors”[19], thereby producing an ELAN with a delayed latency, we analysed ERPs from the following 300–500 ms time window. We found a significant negativity with a posterior distribution, rather than an anterior distribution, for the G-SLI group, but not for the control groups, or the adult subjects (Figure 2). Individual subject analysis reveals the consistency of this negativity across the G-SLI children but not their age matched controls (Figure 2c) (see also supporting Table S2 and Figure S5). This electrical response resembles the component known as the N400, that is associated with semantic processing[12], but not syntactic processing. Interestingly, syntactic violations have also elicited an N400 in adults with an acquired grammatical disorder (aphasia)[20]. Thus, it appears from this study that the G-SLI children were not merely delayed in their response, but were compensating for their impairment in structural syntactic dependencies by using a different neural circuitry associated with semantic mechanisms.

**Figure 3:**
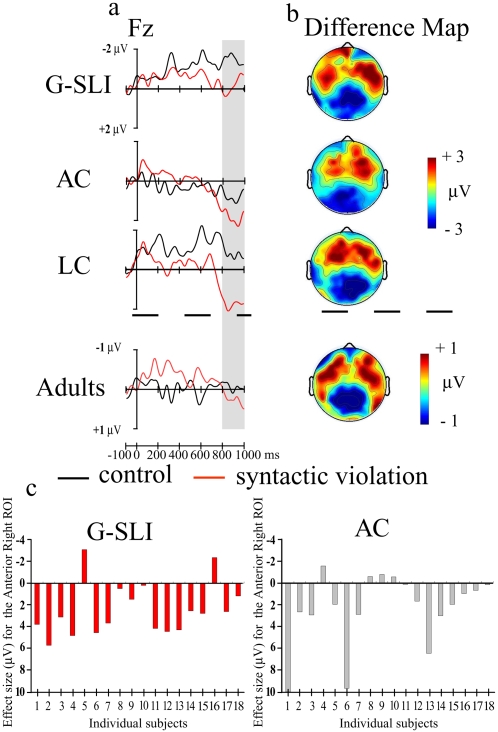
Reanalysis component (P600, 800–1000 ms–grey area) elicited for the syntactic violation. *a.* ERP waveforms, for each group from Fz (frontal electrode). *b.* Scalp distribution of the differences between the violation minus control sentences for each group. The syntactic violation elicited a positivity distributed on anterior regions with a maximum on the right sites for the G-SLI participants (Condition×Caudality: *F*_2,34_ = 15.64, *p*<.0001, Condition×Caudality×Hemisphere *F*_2,34_ = 6.39, *p*<.005); and on anterior regions for the age controls (Condition×Caudality: *F*_2,34_ = 8.93, *p*<.003), language controls (Condition×Caudality: *F*_2,38_ = 17.54, *p*<.001), and adults (Condition×Caudality: *F*_2,38_ = 9.61, *p*<.003). *c.* Effect sizes for individual G-SLI children and their age controls. Effect size: mean amplitude difference (violation minus control) in the Anterior Right ROI within the 800–1000 ms temporal window. We plot Negativity upward.

To assess whether the G-SLI children's deficit extends to other neural correlates that are elicited by the same syntactic violations to the same words, we analysed the time window between 800–1000 ms (Figure 3). These neural correlates are associated with (secondary) re-analysis of the structure, rather than initial structural syntactic processing[10], [11], [13]. Analysis revealed a significant positive electrical response in all groups (overall ANOVA: Condition×ROI (*F*_8, 424_ = 21.81, *p*<.0001, but no main effect of Group, (*F*_2,53_ = 1.33, *p*>.27), or interaction with Group (*p*>.68). This response, in this time window[15], [16] is characteristic of the P600 component, associated with such re-analysis or syntactic integration. The brain maps (Figure 3b) show that it is distributed on the anterior regions of the scalp for the age and language controls, and is equally distributed on the anterior sites for the adults. For the G-SLI group it is also significant on both anterior sites but, interestingly, shows maximum amplitude on the right. This time, individual analysis reveals in both individual G-SLI and age control children a consistent positive electrical response (Figure 3c) (see also supporting Figure S6). This frontal distribution (cf. the centroparietally distributed P600[21]) is commensurate with previous research in adults where, as in this study, the sentence structure at the point of measurement is unexpected, rather than ungrammatical, *per se* [11], [13], [21]. Further, in contrast to the ELAN which appears to be domain-specific, this frontal P600 is modulated by more general cognitive processes[22], [23], and therefore is likely to reflect domain-general processes. Our results, showing dissociation between the ELAN (missing) and P600 (normal) when processing the same word in a sentence in the G-SLI individuals strongly indicate that these two components reflect different computations in syntactic processing. Whereas fast, automatic grammatical structure processing is missing, later sentence analysis is normal. Thus such dissociation is found not only in the mature adult system[10] and patients with lesions[20], but also in developmental disorders.

**Figure 4:**
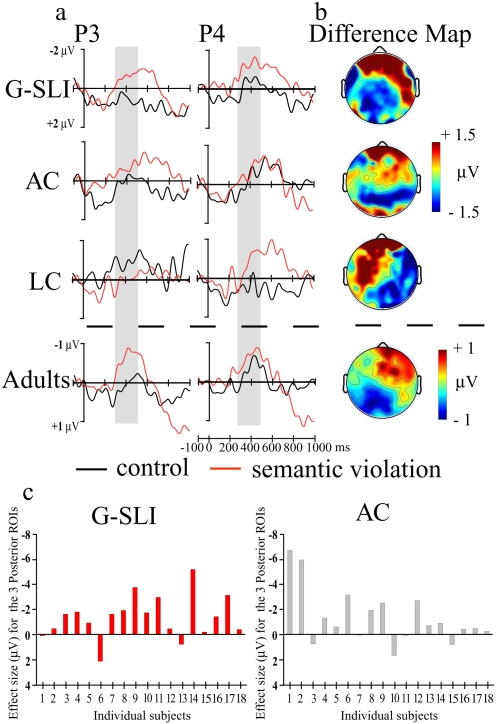
Semantic component (N400, 300–500 ms) elicited for the semantic violation. *a.* ERP waveforms for each group from two posterior electrodes (P3, left and P4 right hemisphere). *b.* Scalp distribution of the differences between the violation minus the control sentences for each group. The semantic violation elicited a posterior negativity for the age controls (Condition×Caudality: *F*_2,34_ = 3.72, *p*<.05) and also the G-SLI group (Condition×Caudality: *F*_2,34_ = 7.15, *p*<.001). This negativity was maximal on the right hemisphere for the language controls (Condition×Hemisphere: *F*_1,18_ = 6.92, *p*<.01), and on the left posterior sites for the adults (Condition×Caudality: *F*_2,38_ = 6.07, *p*<.01, Condition×Hemisphere: *F*_1,19_ = 10.69, *p*<.001). Note that the N400 effect started as early as 100 ms for the G-SLI, age and language controls. *c.* Effect sizes for individual G-SLI children and their age controls. Effect size: mean amplitude difference (violation minus control) in the 3 Posterior ROIs within the 300–500 ms temporal window. We plot Negativity upward.

To investigate the possibility that brain responses to semantic processing in G-SLI were impaired, in Experiment 2 (see materials and methods) we investigated brain responses to sentences with semantic violations (*Barbie bakes the ***people*** *in the kitchen*). We analysed responses to these semantic violations in the time window between 300–500 ms (Figure 4) (see also supporting Table S3). Overall ANOVA revealed a significant effect of group (*F*_2,52_ = 3.56, *p* = .035) but no significant interactions with this factor. The electrical responses in the control groups and the G-SLI participants (Figure 4a) were characteristic of an N400, associated with the brain's detection of semantic anomalies[12]. The group effect was accounted for by differences in the distribution of the N400 in the younger language controls, where we recorded the maximal negativity in the right hemisphere (Figure 4b). In contrast, for the G-SLI children, like the age controls, the N400 was distributed bilaterally in the posterior areas (Figure 4b). Moreover, this N400 is strongly consistent across individual G-SLI children and their age controls (Figure. 4c) (see also supporting Figure S7). Our findings showing differences in the distribution of the N400 according to age concur with previous research[16].

To ensure that the N400 was not elicited later in the G-SLI children due to slow processing, we analysed the peak latency of this response over the posterior areas for the G-SLI children (mean latency 379 ± 20 ms) and the age controls (mean latency 345±20 ms.). ANOVA did not reveal any significant differences between groups (*F*_1,34_ = 1.39, *p* = .24) nor interactions with group (*F*<1). Therefore, neural responses elicited by semantic violations in the G-SLI children and age control children revealed a similar distribution and occurred at a similar millisecond time-point after hearing the beginning of the word.

## Discussion

Overall, the G-SLI subgroup indicates normal semantic processing of language and normal auditory processing speed. Such evidence challenges the view that generally slow or impaired auditory processing causes and maintains grammatical impairment. Note this does not militate against different forms of SLI possibly having different biological and neural instantiations and different developmental outcomes. However, the G-SLI electrophysiological signature reveals a selective developmental deficit in neural circuitry. This neural circuitry is linked to particular aspects of grammar, representing structural syntactic dependency relations, whose evolution is crucial, and possibly unique to the human language faculty[1], [2]. The results argue for grammar being a highly specific, specialised subsystem in the human brain and a particular developmental pathway to this exclusive neural system. The findings indicate that developmental higher cognitive deficits can be selective, which has significant implications for the diagnosis and treatment of SLI. For G-SLI children and perhaps other SLI subgroups too, a relative strength in semantic processing could be targeted to help compensate for their syntactic impairment. The findings provide basic knowledge about the functional architecture of the brain and the development of uniquely human and specialised higher cognitive systems.

## Materials and Methods

### Methods

#### Subjects

We recorded four groups: 18 G-SLI (mean age 14.3, 10–21 years old, 13 males, for selection criteria see[3]), 18 age controls (mean age 14.3, 10–22 years old, 13 males matched with the G-SLI participants on age, sex, laterality and non verbal IQ[24]), 20 language controls (mean age 8.1, 7–9 years old, 11 males, matched with the G-SLI participants on receptive vocabulary[25]) and 20 students from UCL (mean age 23.5, 18–38 years old, 8 males).

#### Experimental Design

In this study we manipulated the animacy property of the first noun following a verb so that in Experiment 1 we created a syntactic violation, and Experiment 2 a semantic violation. Crucially, the syntactic violation relied on a structural syntactic dependency between two non-adjacent words in the sentence, whereas the semantic violation relied on purely lexical semantic restrictions of the preceding verb. Note, technically the syntactic violation was an “unexpectancy” as the following preposition rendered the sentence grammatical. However, pre-testing of the sentences (see below) indicted, that at the critical word to which our EEG recordings were time-locked, the listener would perceive the word as a violation. What is at issue here, is not whether the word is a violation or unexpectancy, but identifying the different functional neural circuitry that is used to detect such a violation/unexpectancy.

#### Experiment 1: Syntactic processing

Here we manipulated the animacy properties of the wh-word (*who* [+animate] vs. *what* [−animate]) in object questions in relation to those of the noun (*clown* [+animate] vs. *ball* [−animate]) following the verb. We constructed questions where the *wh*-word-*noun* pair either matched (syntactic violation) or mismatched (control) (see materials). For questions that contained the animacy match (syntactic violation), a preposition and NP followed the critical noun, making the overall question ungrammatical. For the mismatch pair (control) following the critical noun we added only a preposition, making the overall question grammatical. In doing so, we aimed to focus the participant's attention to the lexical animacy properties of the *wh*-word-*noun* pair: e.g., *Who did Barbie push the* ***clown*** *into the wall?* (animacy match- syntactic violation), *Who did Barbie push the* ***ball*** *into?* (animacy mismatch- control questions). We computed and analysed ERPs from the presentation of the nouns (***clown/ball)*** in the object position. We aimed to identify which neural (and language) systems are incurred when a subject encounters the syntactic violation nouns, rather than the fact that they might later consciously notice the animacy match-ungrammaticality association.

#### Experiment 2: Semantic processing

Using declarative sentences, we manipulated the animacy property of the noun following the verb, in relation to the verb's semantic selection-restrictions; e.g., *bread* [-animate] is a possible noun following the verb, *bake*, (*Barbie bakes the bread in the kitchen*–control sentence) but *people* [+animate] is not *(Barbie bakes the people in the kitchen*–semantic violation).

### Electrophysiological recording and data analysis

We recorded ERPs using the EGI system (128 channels, 250 Hz sampling rate, 0.1–100 Hz). ERPs were re-referenced according to the average reference. Prior to off-line averaging, all single trial waveforms with artefacts were rejected. For Experiment 1, syntactic processing, behavioural responses were ignored because we expected the G-SLI participants to make more errors compared to controls. For Experiment 2, semantic processing, ERPs were averaged from correct behavioural responses only. We rejected one outlier subject from the language control group based on his behavioural responses from Experiment 2. For the syntactic experiment the number of averaged trials did not show any group differences (33 = language control, 34 = G-SLI, 40 = age control, *F*_2,53_ = 2.14, *p*>0.12). For the semantic experiment, a group effect (*F*_2,52_ = 10.71, *p*<0.001) was due to fewer trials being available in the average for the language control (25) compared to the G-SLI (32) and age control group (38).

ERPs (1000 ms epochs) were quantified by mean amplitude measures after the onset of the critical word (direct object noun) for different time windows (TW): the ELAN from 100 to 300 ms, the N400 from 300 to 500 ms and the P600 from 800 to 1000 ms relative to the 100 ms prestimulus baseline. Note, we also analysed the time window from 0 to 100 ms, but found no significant effect for experimental condition or interactions with topographical factors (but see Table S2–S3). Subsequent overall ANOVAs with group (3: G-SLI, age and language controls), condition (2) and ROI (9: the head was divided into nine Regions Of Interest, and for each we computed a single mean amplitude from 6 to 11 electrodes, see supporting Figure S3). We then carried out further ANOVA after rescaling the data[26] to assess differences in scalp topography for each population. Thus, separate ANOVAs (Condition (2): violation, control; Caudality (3): anterior, median, posterior; Hemisphere (2): left, right) for each group as well as the adults were carried out. We report significant effects only when the raw data and the normalized data were both significant. The Greenhouse-Geisser correction was applied to all analyses when evaluating effects with more than one degree of freedom in the numerator.

Ethical approval was granted from the UCL/UCLH ethics committee (01/0150). Signed consent was obtained from participants or their parents/guardians.

## Supporting Information

Data S1 Electrical brain responses to auditory processing in language impaired children
(0.05 MB DOC)

Table S1 Mean latency and amplitude for the N100, P200, and P300 components for the G-SLI and age matched control groups. Lat = Latency; Amp = Amplitude in µV; Mean SD = Mean average Standard Deviation
(0.03 MB DOC)

Table S2 Experiment 1: Syntactic processing: Mean amplitude differences (violation minus control) for the syntactic task within the different windows of interest (0–100 ms, 100–300 ms, 300–500 ms and 800–1000 ms) for each region of interest (ROI), the standard error is shown in italic. We performed a simple ANOVA for each region of interest separately: *** p<.001; ** p<.01; * p<.05. AC: Age Controls, LC: Language Controls.
(0.04 MB DOC)

Table S3 Experiment 2 Semantic processing: Mean amplitude differences (violation minus control) for the semantic task within the different windows of interest (0–100 ms, 100–300 ms, 300–500 ms and 800–1000 ms) for each region of interest (ROI), the standard error is shown in italic. We performed a simple ANOVA for each region of interest separately: *** p<.001; ** p<.01; * p<.05. AC: Age Controls, LC: Language Controls.
(0.03 MB DOC)

Figure S1 Superimposed plot of AEPs for the target and standard tones for the G-SLI and Age control groups.
(0.19 MB JPG)

Figure S2 Mean average map for the periods of interest for the N100, P200 and P300 for the G-SLI and Age control groups.
(0.39 MB JPG)

Figure S3 9 Regions of Interest and the corresponding electrode sites.
(0.38 MB JPG)

Figure S4 Syntactic processing: Effect sizes for individual subjects for the adult and language control (LC) groups in the 100–300 ms temporal window (ELAN). Effect size: mean amplitude differences (violation minus control) in the Anterior Left ROI. Negativity is plotted upwards.
(0.02 MB PDF)

Figure S5 Syntactic processing: Effect sizes for individual subjects for the adult and language control (LC) groups in the 300–500 ms temporal window for the syntactic task. Effect size: mean amplitude differences (violation minus control) in the Posterior Central ROI. Negativity is plotted upwards.
(0.02 MB PDF)

Figure S6 Syntactic processing: Effect sizes for individual subjects for the adult and language control (LC) groups in the 800–1000 ms temporal window (P600). Effect size: mean amplitude differences (violation minus control) in the Anterior Right ROI. Negativity is plotted upwards.
(0.02 MB PDF)

Figure S7 Semantic processing: Effect sizes for individual subjects for the adult and language control (LC) groups in the 300–500 ms temporal window (N400). Effect size: mean amplitude differences (violation minus control) in the 3 Posterior ROIs. Negativity is plotted upwards.
(0.02 MB PDF)

Methods S1 (0.04 MB DOC)
