# Supplementary material for: Electrical Brain Responses in Language-Impaired Children Reveal Grammar-Specific Deficits
Source: PLoS One. 2008 Mar 12;3(3):e1832. doi: 10.1371/journal.pone.0001832 (PMC2268250; doi:10.1371/journal.pone.0001832)
Supplement: Data S1 — Electrical brain responses to auditory processing in language impaired children (0.05 MB DOC) [file pone.0001832.s001.doc]

### Supplementary Data S1

### Auditory Processing

To control for a more basic, lower-level auditory processing deficit, we recorded Auditory Evoked Potentials (AEPs) of G-SLI and age matched control participants to pure tones in a classical auditory oddball paradigm. Auditory processing elicits early electrophysiological responses known as the N100/P200 (or N1/P2) complex. This complex is associated with perceptual detection of a discrete change in the auditory environment. In addition, they elicit a later P300 component that reflects attentional control processes to detect and categorize a specific event.

#### We compared the G-SLI and age-matched control groups’ AEPs to pure tones (50 ms long segments with 10 ms rise and fall time) in an attended odd-ball paradigm. The targets were 2000 Hz tones (high tones) presented 20 % of the time, whereas the standards were 1000 Hz tones (low tones) presented 80 % of the time. The tones were presented through sound speaker at 60-70 dB. The interstimulus interval (ISI) was 800 ms and tones were randomised so that no more than two target stimuli occurred successively. 60 target and 240 standard tones were presented over a seven minute period.

Participants sat in a comfortable chair in an electrically shielded faraday cage approximately 100 cm from the monitor. The children looked at cartoon pictures of animals presented on the monitor (to prevent boredom). Whenever the target (high tone) was presented, they had to press a button on a button box with their dominant hand “as quickly as possible”. An adult sat with each participant during the task to monitor attention to the task and minimise movement artefacts.

### Electrophysiological recordings

The EEGs were recorded continuously at a sampling rate of 250 Hz, with a band pass of 0.1 to 100 Hz from a montage of 128 recording sites with the Geodesic Sensor Net (manufactured by electrical geodesic Inc). The Cz (vertex) electrode was used as the recording reference. The EEG was first divided into segments from 100 ms pre-stimulus to 800 ms post-stimulus. Segments containing vertical EOG (eye blink) or horizontal EOG (eye movement) activity greater than 150 μV were excluded from averaging. This resulted in two sets (target and standard tones) of averaged evoked potentials for each participant. ERPs were re-referenced according to the average reference. ERPs were quantified in relation to the 100 ms pre-stimulus baseline.

### ERP analysis

Three dependent variables were measured: **peak latencies** (highest or lowest peak latency on the curve in ms), **peak amplitudes** (highest or lowest peak relative to the prestimulus baseline in µV), **mean amplitudes** (mean average amplitude within a time window of interest in µV). These were defined as follows:

**Peak latencies and peak amplitudes for three selected electrodes Fz, Cz, Pz**

⁫ N100: largest negative-going deflection in the time window 70-190 ms

⁫ P200: largest positive-going deflection in the time window 120-220 ms

⁫ P300: largest positive-going deflection in the time window 220-700 ms

**Mean amplitudes for selected electrodes grouped into Regions Of Interest**

A description of the ROIs are provided in the supplementary Methods S1 and Figure S-3.

⁫ N100: 80-140 ms

⁫ P200: 140-200 ms

⁫ P300: 250-650 ms

The resulting AEPs from both groups showed an N100 after about 120 ms followed by a P200 around 180 ms for both tones, and a P300 component for the target tones (Figure S-1). Note the Greenhouse-Geisser correction was applied to all analyses when evaluating statistical effects with more than one degree of freedom in the numerator

**Peak Latency**

To assess whether the G-SLI children were slow auditory processors, we first analysed the peak latency of each component according to the definitions above (see supplementary Table S-1, below).

Analysis revealed a similar pattern of results for the N100, P200 and the P300 for the two groups. There were no significant effects of group (N100, *F*_1, 34_=.38, *p*=.53; P200, *F*_1, 34_=1.27, *p*=.26; P300, *F*_1, 34_=.08, *p*=.76), but significant effects of condition (N100, *F*_1, 34_=10.32, *p*=.002; P200, *F*_1, 34_=5.17, *p*=.02; P300, *F*_1, 34_ =5.6, *p*=.02) and electrodes for the N100 (*F*_2, 68_=9.67, *p*=.0002) and the P300, *F*_2, 68_=22.20, *p*<.0001). There were no significant interactions with group for any of the components (*F*<1). The G-SLI children and Age controls showed a shorter latency for the target tones (N100, 117.7 ms; P200, 172.1 ms; P300, 446.2 ms) than the standard tones (N100, 125.4 ms; P200, 179.5 ms; P300, 491.6 ms). Moreover, for the N100 and P300, for both conditions, the latency of the N100 is shortest at Pz (N100, 111.8 ms; P300, 405.4 ms) compared to Fz (N100, 128.3 ms, *p*<.00006; P300, 548.7 ms, *p*<.00001) or Cz (N100, 124.5 ms, *p*=.0001; P300, (452.6 ms, *p*=.03). Thus, electrical brain responses associated with perceptual detection of a discrete change in the auditory environment (N100/P200), and attentional control processes to detect and categorise a specific auditory event (P300), were as fast for the G-SLI children as their age matched controls. These results revealed no evidence that G-SLI children were slow in basic auditory processing which could have affected their processing of sentences.

### Peak Amplitude

The second set of analyses investigated the peak amplitudes of the three components (supplementary Table S-1). Increased amplitude of components is often associated with increased effort to process information or carry out a task. Therefore, such amplitude increases if found in our study could reveal a subtle deficit in auditory processing.

Analysis for each of the components revealed a similar pattern of results for both groups. The Peak amplitude show no significant effects of group (N100, *F*_1, 34_=.86, *p*=.36; P200, *F*_1, 34_=2.72, *p*=.10; P300, *F*_1, 34_=0.27, *p*=.60), a significant effect of condition (N100, *F*_1, 34_=6.36, *p*=.01; P200, *F*_1, 34_=7.54, *p*=.009; P300, *F*_1, 34_=93.7, *p*< .0001) and electrodes (N100, *F*_2, 68_=17.69, *p*<.00001; P200, *F*_2, 68_=53.21, *p*<.00001; P300, *F*_2, 68_=66.15, *p*<.0001), but no interactions with group for any of the analyses (*F*<1). Our results show that the amplitude for the target tones (N100, - 2.82 µV; P200, 1.71 µV; P300, 5.91 µV) is larger compared to the standard tones (N100, -1.96 µV, P200, 0.93 µV; P300, 2.02 µV). For the N100, for both condition, the amplitude is larger at Fz (- 3.68 µV) compared to Cz (- 2.19 µV, *p*<.0004) or Pz (- 1.30 µV, *p*<.001); whereas for the P200 it is larger at Pz (3.10 µV) compared to Fz (- 1.18 µV, *p*<.0001) or Cz (2.05 µV, *p*<.01).

For the P300, analysis revealed a significant interaction between condition and electrodes (*F*_2, 68_=29.99, p<.0001), that reflected a larger amplitude for the target at Pz (9.9 µV) compared to Fz (1.1 µV, *p*<.0001) and Cz (-6.6 µV, *p*<.0001; whereas for the standard tone the amplitude was equally distributed on the Pz and Cz electrodes (Fz: 0.86 µV, Cz: 2.3µV, Pz: 2.90 µV).

Importantly, these results reveal no evidence of any difference in amplitude for any component between the G-SLI and the Age-Matched control groups.

### Mean amplitudes

The third set of analyses was carried out on normalized data and took into consideration the whole montage of 128 electrodes. This enabled us to assess whether there were any topographical differences between the groups in the scalp distribution of the electrical responses. To do this we investigated interactions between the factor group and condition or ROI. The brain maps (Fig. S-2) show that the distribution of the three components is similar for the G-SLI and age control groups. Statistical analysis revealed no significant interaction between groups and condition or ROI for any of the components. For both groups the N100 component shows maximum amplitude on the frontal electrodes (Caudality, *F*_2, 68_=16.77, *p*<.0001), whereas for the P200 the maximum amplitude is on the central regions (Caudality, *F*_2, 68_=112.1, *p*<.0001) and is larger for the target tones compared to the standard tones (Condition x Caudality, *F*_2, 68_=7.7, *p*<.0001). For the P300, both groups produced a larger amplitude for the target tones compared to the standard tones (Condition, *F*_1, 34_=14.19, *p*<.0001), which was larger on the posterior left hemisphere for the target compared to the standard tones (Condition x Caudality x Hemisphere, *F*_2, 68_=8.25, *p*<.0001) (see Figure S2)

In sum, we discovered that children with G-SLI have age-appropriate waveforms for the N100, P200 and the P300 components (latency, amplitude, distribution on the scalp). Our results reveal that G-SLI children have normal auditory processing during the discrimination of pure tones.
