# Supplementary material for: Electrical Brain Responses in Language-Impaired Children Reveal Grammar-Specific Deficits
Source: PLoS One. 2008 Mar 12;3(3):e1832. doi: 10.1371/journal.pone.0001832 (PMC2268250; doi:10.1371/journal.pone.0001832)
Supplement: Table S1 — Mean latency and amplitude for the N100, P200, and P300 components for the G-SLI and age matched control groups. Lat = Latency; Amp = Amplitude in µV; Mean SD = Mean average Standard Deviation (0.03 MB DOC) [file pone.0001832.s002.doc]

**N100** Age Control G-SLI

______________________________________________________

Lat Amp Lat Amp

Mean *SD* Mean *SD* Mean *SD* Mean *SD*

_______________________________________

Standard *Fz* 130.2 *25.9* -2.7 *1.5* 135.5 *17.2* -3.3 *1.8*

*Cz* 122.8 *16.4* -1.7 *1.3* 131.8 *23.2* -2.0 *1.5*

*Pz* 119.4 *23.7* -1.0 *1.0* 112.9 *21.4* -0.9 *1.5*

Target *Fz* 120.5 *27.4* -3.8 *2.2* 127.2 *20.1* -4.7 *3.0*

*Cz* 120.0 *21.9* -2.2 *2.5* 123.7 *31.0* -2.7 *2.1*

*Pz* 108.2 *20.5* -1.7 *3.3* 107.1 *22.3* -1.4 *3.1*

**P200** Age Control G-SLI

______________________________________________________

Lat Amp Lat Amp

Mean *SD* Mean *SD* Mean *SD* Mean *SD*

_______________________________________

Standard *Fz* 166.1 *24.9* -0.8 *2.0* 174.3  *23.8* -1.2 *1.8*

*Cz* 183.3 *28.9* 1.2 *1.8* 190.1 *22.8* 2.1 *1.4*

*Pz* 183.4 *29.3* 1.7 *1.2* 179.7  *40.4* 2.6 *1.4*

Target *Fz* 162.1 *32.1* -1.8 *2.1* 182.3  *26.8* -0.7 *2.5*

*Cz* 171.1 *28.2* 1.9 *2.4* 177.8  *25.1* 2.9 *3.7*

*Pz* 168.1 *29.3* 3.4 *2.4* 170.6  *33.3* 4.4 *4.2*

**P300** Age Control G-SLI

______________________________________________________

Lat Amp Lat Amp

Mean *SD* Mean *SD* Mean *SD* Mean *SD*

_______________________________________

Target *Fz* 495.2 *151.8* 1.1 *2.4* 532.2 *155.6* 1.1 *2.8*

*Cz* 416.0 *106.7* 6.4 *3.5* 399.1 *103.1* 6.9 *4.4*

*Pz* 401.7 *95.8* 9.5 *4.2* 433.1 *124.4* 10.3 *4.6*
