# Supplementary material for: Electrical Brain Responses in Language-Impaired Children Reveal Grammar-Specific Deficits
Source: PLoS One. 2008 Mar 12;3(3):e1832. doi: 10.1371/journal.pone.0001832 (PMC2268250; doi:10.1371/journal.pone.0001832)
Supplement: Table S2 — Experiment 1: Syntactic processing: Mean amplitude differences (violation minus control) for the syntactic task within the different windows of interest (0–100 ms, 100–300 ms, 300–500 ms and 800–1000 ms) for each region of interest (ROI), the standard error is shown in italic. We performed a simple ANOVA for each region of interest separately: *** p<.001; ** p<.01; * p<.05. AC: Age Controls, LC: Language Controls. (0.04 MB DOC) [file pone.0001832.s003.doc]

Anterior Median Posterior

Left Central Right Left Central Right Left Central Right

________________________ ________________________ ________________________

**0-100 ms**

Adults -0.04 *0.12* -0.07 *0.11* -0.08 *0.11* -0.01 *0.10* 0.23 *0.10* 0.10 *0.11* -0.04 *0.11* 0.07 *0.13* 0.12 *0.12*

AC -0.33 *0.27*  -0.47 *0.24* 0.15 *0.23*  -0.34 *0.17* -0.44 *0.22* -0.01 *0.21* -0.22 *0.25*  -0.23 *0.24*  0.20 *0.22*

LC -0.47 *0.28* 0.06 *0.37* 0.60 *0.35* -0.18 *0.29*  0.32 *0.34* 0.97 *0.22* ******  -0.78 *0.48* -1.00 *0.38* -0.25 *0.26*

G-SLI 0.14 *0.23* 0.08 *0.29* 0.32 *0.22* 0.08 *0.22* 0.28 *0.22* 0.12 *0.19*  -0.19 *0.29* -0.18 *0.26* -0.04 *0.19*

**100-300 ms**

Adults -0.38 *0.13* ***** -0.48 *0.14* ***** -0.24 *0.14* -0.03 *0.11* 0.23 *0.14* 0.34 *0.13*  0.27 *0.13* 0.56 *0.14* ****** 0.47 *0.13* ******

AC -0.96 *0.37* ******* -0.69 *0.36* ***** 0.34 *0.37* -0.36 *0.20* 0.26 *0.27* 0.65 *0.25* 0.00 *0.27* 0.29 *0.45*  0.88 *0.35**

LC -0.73 *0.45* ***** 0.64 *0.52* 0.81 *0.39* ***** -0.71 *0.31* ***** 0.22 *0.35* 1.00 *0.31* ****** -0.85 *0.51* -1.53 *0.49* ***** -0.69 *0.41*

G-SLI 0.13 *0.29* 0.12 *0.32*  0.37 *0.33* 0.35 *0.27* -0.55 *0.34* -0.09 *0.28*  -0.01 *0.29*  -0.75 *0.29* ***** -0.51 *0.34*

**300-500 ms**

Adults -0.30 *0.17*  -0.37 *0.20* -0.08 *0.19*  -0.10 *0.13* 0.08 *0.16* 0.25 *0.13*  0.15 *0.16* 0.34 *0.20*  0.30 *0.17*

AC -0.56 *0.41* 0.62 *0.47* 0.15 *0.45* -0.27 *0.29* 0.08 *0.24* 0.59 *0.23* ***** 0.23 *0.38* 0.65 *0.50* 1.22 *0.43* *****

LC -0.75 *0.50* 0.11 *0.60*  -0.18 *0.53*  -0.52 *0.39*  0.66 *0.35* 0.52 *0.38* -0.12 *0.52* -0.32 *0.50* -0.81 *0.46*

G-SLI 0.02 *0.44* 0.24 *0.48*  0.74 *0.38 ** 0.43 *0.35*  -0.20 *0.41* 0.17 *0.31* -0.22 *0.31*  -1.07 *0.40* ***** -0.74 *0.38* *****

**800-1000 ms**

Adults 0.70 *0.22* ***** 0.35 *0.21* 0.78 *0.22* ****** 0.11 *0.19* -0.26 *0.26*  0.29 *0.19*  -1.02 *0.23* *******  -1.51 *0.27* ******* -0.82 *0.25* ******

AC 1.63 *0.69* ***** 1.09 *0.71* 2.32 *0.59* ******  0.91 *0.38* *****  0.53 *0.41*  1.60 *0.45 ** -1.48 *0.48* ***** -2.34 *0.66* ****** -0.93 *0.58* *****

LC 2.93 *0.65* ****** 2.15 *0.69* ***** 2.02 *0.49* ****** 0.77 *0.37* 0.04 *0.41* 0.39 *0.43* -1.54 *0.52* ***** -3.01 *0.56* ****** -3.02 *0.56* ******

G-SLI 1.33 *0.58* 1.14 *0.78* 2.47 *0.41* ******* 1.22 *0.40* ***** -0.74 *0.61* 0.67 *0.40* -1.25 *0.44* ***** -2.92 *0.67* ****** -2.24 *0.55* ******
