# Supplementary figures and images for: Electrical Brain Responses in Language-Impaired Children Reveal Grammar-Specific Deficits
Source: PLoS One. 2008 Mar 12;3(3):e1832. doi: 10.1371/journal.pone.0001832 (PMC2268250; doi:10.1371/journal.pone.0001832)

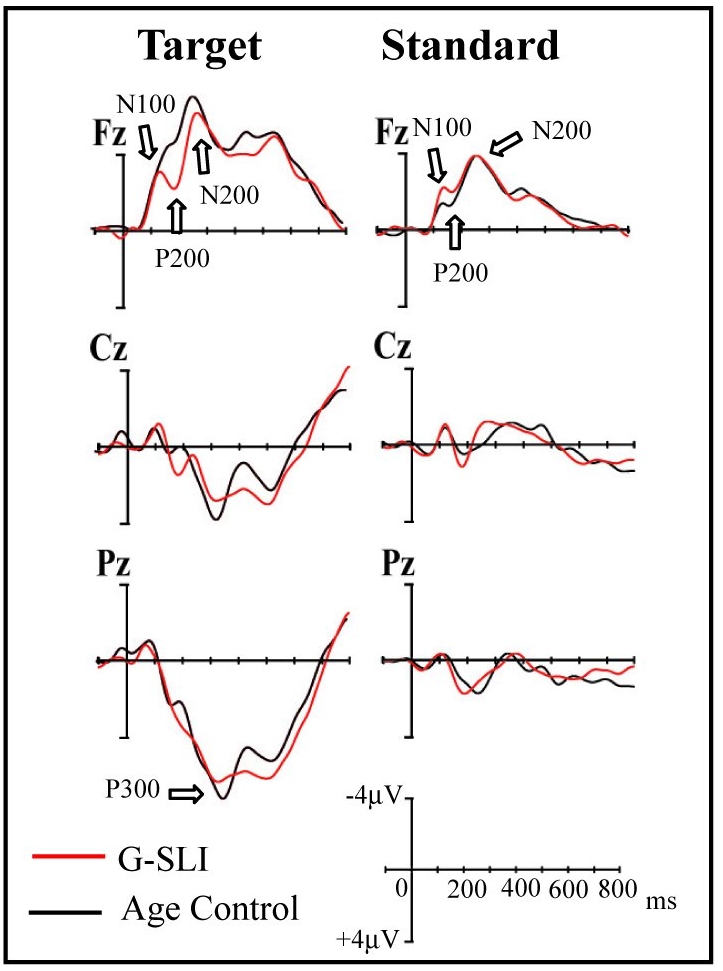

Supplement: Figure S1 — Superimposed plot of AEPs for the target and standard tones for the G-SLI and Age control groups. (0.19 MB JPG) [file pone.0001832.s005.jpg]

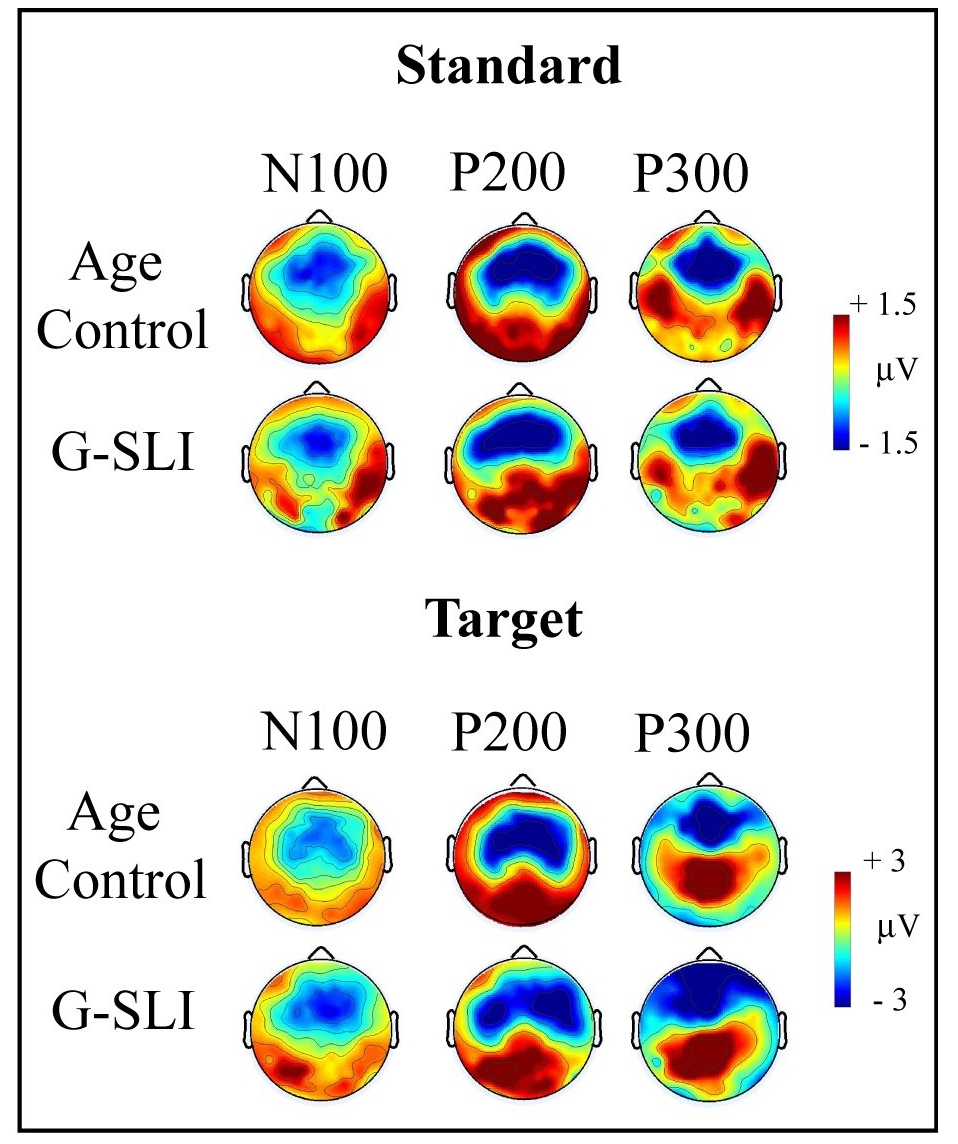

Supplement: Figure S2 — Mean average map for the periods of interest for the N100, P200 and P300 for the G-SLI and Age control groups. (0.39 MB JPG) [file pone.0001832.s006.jpg]

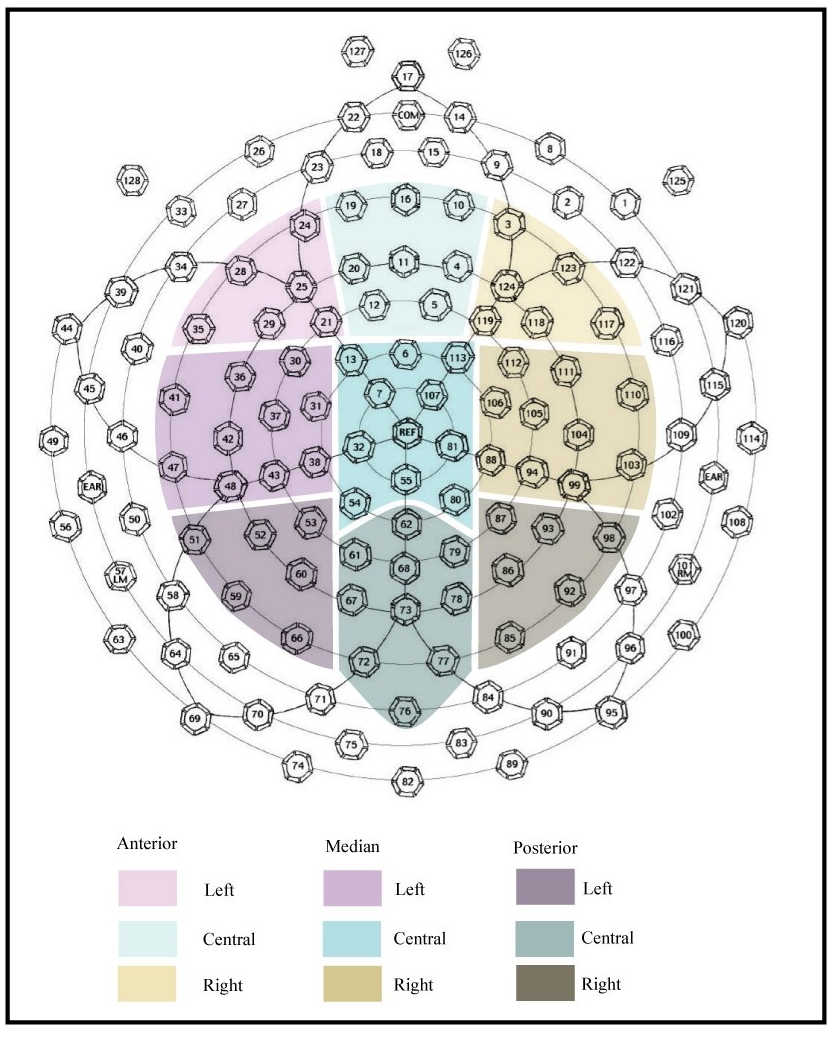

Supplement: Figure S3 — 9 Regions of Interest and the corresponding electrode sites. (0.38 MB JPG) [file pone.0001832.s007.jpg]

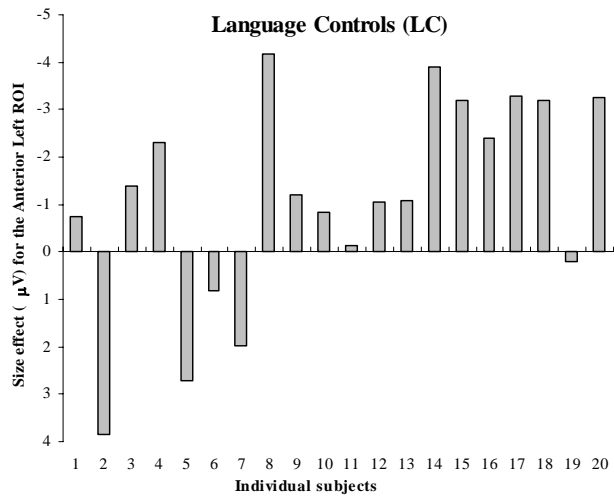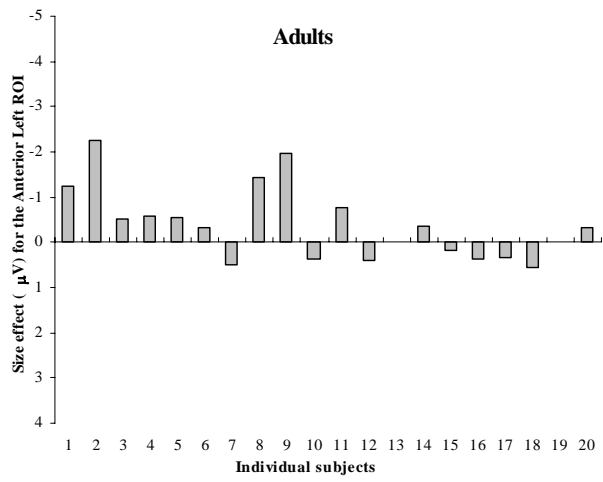

Supplement: Figure S4 — Syntactic processing: Effect sizes for individual subjects for the adult and language control (LC) groups in the 100–300 ms temporal window (ELAN). Effect size: mean amplitude differences (violation minus control) in the Anterior Left ROI. Negativity is plotted upwards. (0.02 MB PDF) [file pone.0001832.s008.pdf]

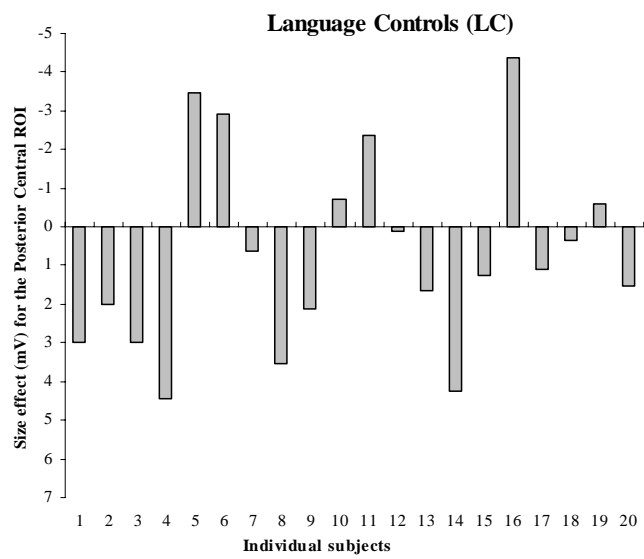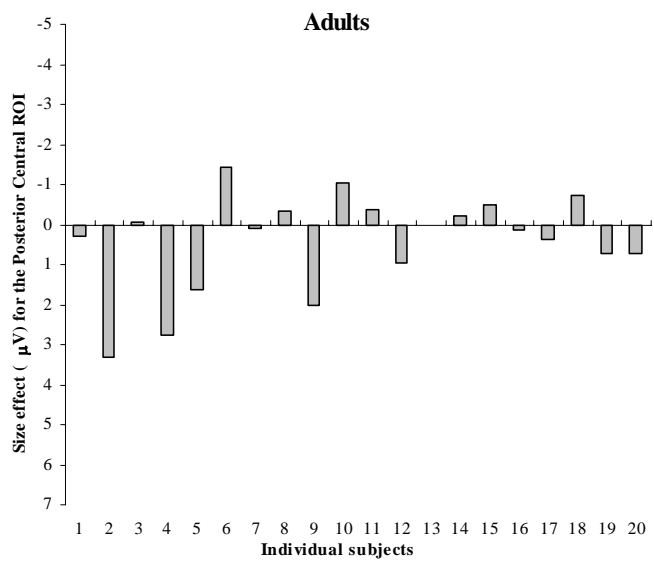

Supplement: Figure S5 — Syntactic processing: Effect sizes for individual subjects for the adult and language control (LC) groups in the 300–500 ms temporal window for the syntactic task. Effect size: mean amplitude differences (violation minus control) in the Posterior Central ROI. Negativity is plotted upwards. (0.02 MB PDF) [file pone.0001832.s009.pdf]

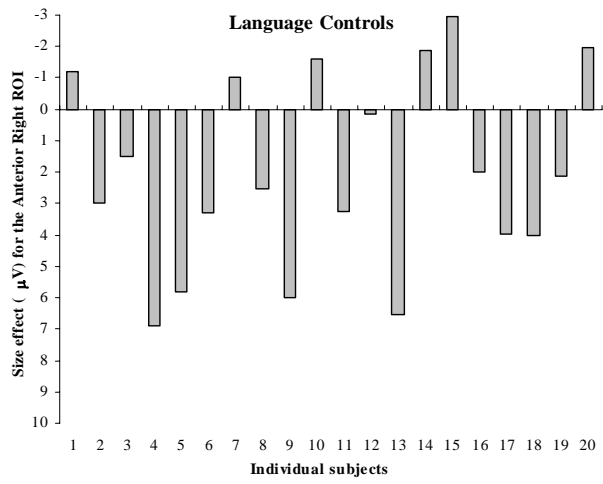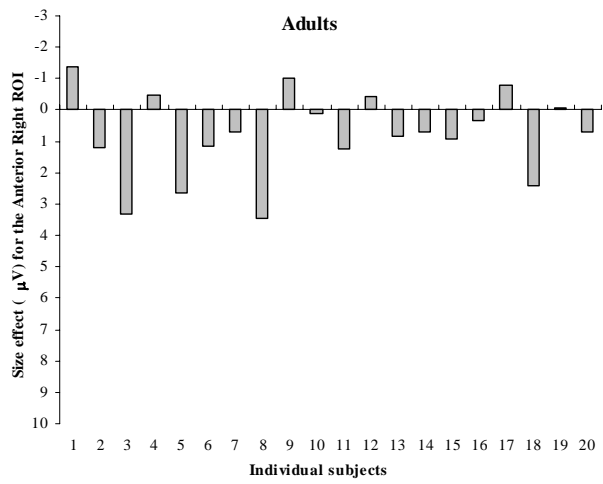

Supplement: Figure S6 — Syntactic processing: Effect sizes for individual subjects for the adult and language control (LC) groups in the 800–1000 ms temporal window (P600). Effect size: mean amplitude differences (violation minus control) in the Anterior Right ROI. Negativity is plotted upwards. (0.02 MB PDF) [file pone.0001832.s010.pdf]

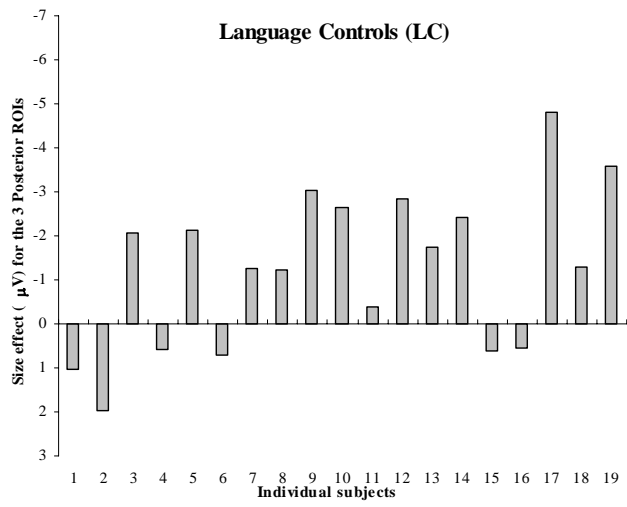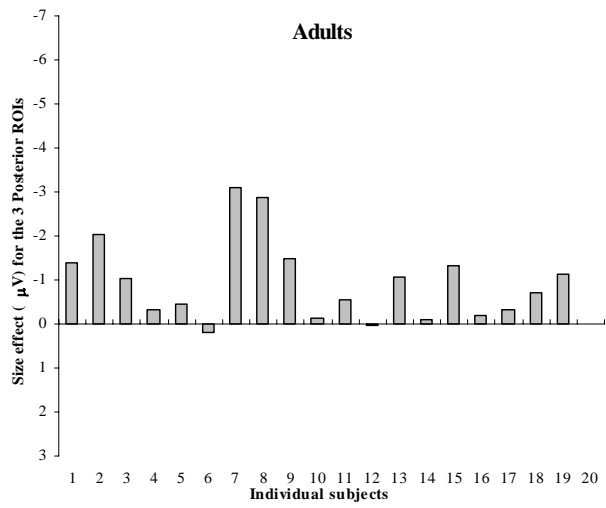

Supplement: Figure S7 — Semantic processing: Effect sizes for individual subjects for the adult and language control (LC) groups in the 300–500 ms temporal window (N400). Effect size: mean amplitude differences (violation minus control) in the 3 Posterior ROIs. Negativity is plotted upwards. (0.02 MB PDF) [file pone.0001832.s011.pdf]
