# Supplementary material for: Electrical Brain Responses in Language-Impaired Children Reveal Grammar-Specific Deficits
Source: PLoS One. 2008 Mar 12;3(3):e1832. doi: 10.1371/journal.pone.0001832 (PMC2268250; doi:10.1371/journal.pone.0001832)
Supplement: Methods S1 — (0.04 MB DOC) [file pone.0001832.s012.doc]

# Supplementary Methods S1

## 2.1 Experimental Materials: Experiment 1: Syntactic processing

160 sentences were prepared. The noun phrases (NPs) in the subject position of the questions consisted of 80 characters/personalities that were highly familiar to children and teenagers (e.g. Homer, Marge, and other cartoon characters and personalities). Each of these subject NPs was heard twice, once in each condition, but with a different verb and target object noun. 80 transitive verbs were used which were repeated once for each participant, but presented with a different subject and object NP in the two conditions (syntactic violation, control). The repetition order (violation, control) was controlled. The object NPs consisted of a determiner (*the, his, her*) and 160 nouns – 80 animate, and 80 inanimate. The nouns and verbs were controlled for age-of-acquisition (under 6 years), frequency, number of syllables and imageability between the two conditions^1-5^. None of these object NPs was repeated.

From this first list, we constructed a second list in which the wh-word was exchanged so that *who* sentences became *what* questions, and the preposition phrase following the target noun was changed accordingly—producing a balanced quartet of questions: For example,

**List 1**

*Who did Barbie push the* ***clown*** *into the wall?* (animacy match- syntactic violation),

*Who did Alice in Wonderland push the* ***ball*** *into?*(animacy mismatch- control)

**List 2**

*What did Barbie push the* ***clown*** *into?* (animacy mismatch- control)

*What did Alice in Wonderland push the* ***ball*** *into the wall?* (animacy match- syntactic violation)

Subjects heard either list 1 or 2.

### 2.1.1 Pre-testing the animacy property of direct object nouns in questions.

Animacy properties of words enter into overt grammatical processes in languages, such as Romance and American-Indian languages^6^. For example, two words (*noun, verb*) in a sentence might have to agree in animacy properties, in the same way as in English words overtly agree in person (*He jump****s*** vs. *I jump)* and number *(A cat*vs. S*ome cat****s***). Thus, the grammaticalisation of properties of words such as, person and number, as well as animacy, is generally found in languages.

We looked to see whether such animacy properties also affect children and adults’ language in English in the pre-tests of our experimental questions.

Two forms of questions in English are: a) wh-questions; and b) yes-no questions.

a) wh-question: *Who did Barbie push the car into______?*

b) yes-no question: *Did Barbie push the car into the clown?*

There is a direct structural relation between these two types of questions. When the wh-word is present, it encodes one of the “arguments” (participants or things) associated with the verb or other predicate (e.g., preposition, *in, with*) in the question. Thus the wh-word has a structural syntactic relation with the position that is normally filled by this noun. Specifically, the “w*ho”* wh-word encodes for an animate and the “*what”*  wh-word for an inanimate property of the noun associated with a verb or other predicate. When the wh-word is not included in the question, the noun phrase that the wh-word is standing for has to be present. The example a) above, shows that in wh-questions, the “*who”* stands for *“the clown”* which is present at the end of the question in the yes-no question (b).

We hypothesised that when the participant heard the determine in our experimental questions, he/she would already “know” that the Noun Phrase (NP) was filled, and therefore must posit that the wh-word was structurally related to another (missing) NP in the question. In view of this, we checked to see when participants completed questions containing a determiner (*the his)* but missing direct object noun, whether the animacy property of this noun would match or mismatch those of the wh-word in the wh-questions, or the indirect object noun in the yes-no questions. Such a process would require checking of the animacy properties between words—specifically the wh-word and the noun, or between the verb and the direct and indirect object nouns. Such syntactic checking in wh-questions, perhaps prior to even hearing the noun, could result in an expectation that the object noun if present would have certain lexical-grammatical properties or features (e.g., it would be a noun rather than a verb, and be inanimate rather than animate).

Based on these properties of question, we pre-tested the questions used in Experiment 1 in two ways.

***Pre-testing adults***

In the first pre-test 21 adults (university undergraduates) were asked to complete 160 written wh-questions used in Experiment 1. The questions were exact copies of those questions, but stopped after the determiner and before the critical noun. For example,

*c) Who did Barbie push the………*

*d) What did Alice in Wonderland push the……*

Participants were asked to complete the questions with the first words that came into their heads. Responses to the first noun in the object position were coded according to whether they were animate (specifically, they could be the answer to a “*who”* question), or whether they were inanimate (specifically, they could be the answer to a *“what”* question). Analysis revealed that participants were significantly more likely to continue the questions that started with a *who* wh-word with an inanimate noun compared to when the wh-word was w*hat,* and vice versa (*p*<.0001). Therefore, adults do not expect that if the first noun phrase after the verb is filled in a wh-question, that it will have the same animacy properties as that of the wh-word. Therefore, adults avoid using a noun phrase that could be a potential answer to the question.

***Pre-testing children and teenagers***

In the second pre-test of the questions, we used this structural syntactic relation between a wh-word and the noun, and the contrasting animacy properties of *who* and *what,* to construct incomplete yes-no questions for the children and teenagers to complete. Thus, using the wh-question from Experiment 1, we constructed yes-no questions where the final noun would be either animate (matching the animacy properties of the “*who”* wh-word) or inanimate (matching the animacy properties of a “*what”* wh-word). The direct object noun was omitted as shown in the examples below.

*e) Did Barbie push the_____ into the clown?*

*f) Did Alice in Wonderland push the _____ into the car?*

Test-response forms were constructed with 6 yes-no questions; 3 from the *“who”* question list and three from the *“what”* question lists. The questions were written in a random order on the test-response forms. Written instructions asked the participant to complete the question. They were told that there was no right or wrong answer, but they were to respond with the first word they could think of.

All 320 questions to be used in Experiment 1 were used in the pre-testing. Thereby we provided exact pairs of wh-yes/no questions with all words matching the test questions including the determiner (the, my, his) before the missing word.

776 school-aged children, evenly distributed over the ages 7 to 19 years, from schools in London participated. Participants were given a form, each with 6 yes-no questions. For each of the 80 verbs used in the experiment we collected approximately 30 who-question-related responses and 30 what-question-related responses (Total 4,650 responses). The direct noun responses were coded according to whether they were animate or inanimate, as in the adult pre-test. Analysis revealed a highly significant effect of animacy of the nouns according to whether the indirect object noun was animate or not. Significantly fewer nouns were animate when the final noun was animate than when the final noun was inanimate (t_765_=10.69, *p*<.0001). There was no significant effect of age when the final noun was animate (r_764_=.019, *p*=.6). In contrast, a small effect of age was found when the final noun was inanimate, with more animate noun responses with increasing age (r_764_=.100, *p*=.001). However, this small effect accounts for only 1% of the variance in the data set.

These data indicate that children and adults process the animacy properties of nouns and wh-words in questions.

## 2.2 Experimental Materials: Experiment 2 Semantic processing

In this experiment each participant listened to 160 declarative sentences (80 per condition).

The subject NPs consisted of the same 80 characters/personalities that were used in Experiment 1. Each NP was repeated once in the two conditions. 80 verbs were used that were different from those used in Experiment 1. Each verb was repeated once but with a different subject and object NPs, balanced across the two conditions (control and semantic violation). 160 nouns were used in the object NP position. None of the nouns were repeated, or appeared in Experiment 1. As in Experiment 1, the verbs and object NPs were controlled for age-of-acquisition, frequency, number of syllables, imageability and animacy across the conditions.

## 2.3 Acoustic recording of stimuli

The stimuli were digitally recorded in the UCL anechoic chamber by two native English female speakers at a normal-slow speaking rate. Following a behavioural pilot study, for Experiment 1 the wh-word at the beginning of the questions was slightly stressed to ensure that the participants paid attention to the wh-word. For the sentences in the syntactic violation condition, speakers spoke the sentence twice, once with the target wh-word (*who/what*) and once where the initial wh-word was replaced by *where* or *when*, making the sentence grammatical (*When did Barbie push the* ***clown*** *into the wall).*  The target wh-word from the syntactic violation condition was then spliced into the grammatically correct sentence. This was done to avoid any prosodic abnormalities which tended to occur when the sentences were un-grammatical. The final sentence lists were balanced for the quartet wh-word, speaker, and 2 conditions. Analysis revealed no acoustic differences before the critical word (intensity, pitch and length) between the conditions for either experiment.

## 2.4 Procedure

Presentation order of experiments was balanced across participants within groups.

Each experiment was split into 3 blocks of 6 minutes. The participants judged the appropriateness of the sentences (motor delayed response).

## 2.5 Supplementary Figure S3 Regions of Interest

See Figure S3

### Additional References

1. Masterson, J. & Druks, J. Description of a set of 164 nouns and 102 verbs matched for printed word frequency, familiarity and age-of-acquisition. *Journal of Neurolinguistics* **11**, 331-354 (1998).

2. Fenson, L. et al. *The MacArthur Communicative Development Inventories: User's Guide and Technical Manual* (Singular Publishing Group, San Diego, 1993).

3. Coltheart, M. The MRC Psycholinguistic Database. *Quarterly Journal of Experimental Psychology* **33A**, 497-505 (1981b).

4. Bird, H., Franklin, S. & Howard, D. Age of Acquisition and imageability ratings for a large set of words, including verbs and function words. *Behavioral Research Methods, Instruments and Computers* **33**, 73-79 (2001).

5. Druks, J. & Masterton, J. *An Object and Action Naming Battery* (Psychology Press, London, 2000).

6. Baker, M. C. *The Polysynthesis Parameter.* (Oxford University Press, New York, 1996).
